# Histopathological Types, Clinical Presentation, Imaging Studies, Treatment Strategies, and Prognosis of Posterior Pituitary Tumors: An Updated Review

**DOI:** 10.3390/jcm14134553

**Published:** 2025-06-26

**Authors:** Pedro Iglesias

**Affiliations:** 1 Department of Endocrinology and Nutrition, Hospital Universitario Puerta de Hierro Majadahonda, C. Joaquín Rodrigo, 1, 28222 Majadahonda, Madrid, Spain; piglo65@gmail.com; 2 Instituto de Investigación Sanitaria Puerta de Hierro Segovia de Arana, 28222 Majadahonda, Madrid, Spain

**Keywords:** posterior pituitary tumors, pituicytoma, granular cell tumor, spindle cell oncocytoma, sellar ependymoma

## Abstract

Posterior pituitary tumors (PPTs) are rare, non-neuroendocrine neoplasms derived from pituicytes of the neurohypophysis or infundibulum. According to the 2025 WHO classification, PPTs comprise four distinct but related low-grade entities: pituicytoma, granular cell tumor of the sellar region, spindle cell oncocytoma, and ependymal pituicytoma. All share nuclear TTF-1 expression, confirming their common origin, but differ in morphology, immunophenotype, and ultrastructure. Histologically, pituicytomas consist of bipolar spindle cells in fascicles; granular cell tumors show polygonal cells with PAS-positive, diastase-resistant cytoplasmic granules; spindle cell oncocytomas display oncocytic change and abundant mitochondria; and ependymal pituicytomas exhibit perivascular pseudorosettes and EMA positivity in apical or dot-like patterns. Immunohistochemically, all are S100 and vimentin positive, and negative for pituitary hormones and lineage-specific transcription factors. Clinically, PPTs are typically non-functioning but may be associated with corticotroph or somatotroph hyperfunction. Imaging features are nonspecific. Surgical resection is the treatment of choice, although hypervascularity and adherence—especially in spindle cell oncocytomas—can hinder complete excision. Radiotherapy is reserved for recurrences. Molecular analyses reveal recurrent alterations in MAPK/PI3K pathways (e.g., HRAS, BRAF, FGFR1, NF1, TSC1) and suggest a shared histogenesis. Copy number imbalances correlate with reduced progression-free survival in some subtypes. Despite a generally favorable prognosis, recurrence—particularly in spindle cell oncocytomas—necessitates long-term follow-up. The WHO 2025 update provides a unified framework for classification, diagnosis, and prognostic stratification of these rare tumors.

## 1. Introduction

The neurohypophysis, also known as the posterior pituitary, is the posterior portion of the pituitary gland. Unlike the adenohypophysis, which originates from Rathke’s pouch—an invagination of the primitive oral ectoderm with an epithelial and glandular origin that enables it to synthesize and secrete various hormones—the neurohypophysis derives from neural ectoderm and develops from an evagination of the diencephalon, specifically from the infundibulum of the hypothalamus. As it originates from neural tissue, it does not produce hormones but rather stores and releases those synthesized in the hypothalamus [1].

The neurohypophysis is formed by axons of hypothalamic neurons located in the supraoptic and paraventricular nuclei of the hypothalamus; Herring bodies, which function as hormone storage vesicles prior to release; pituicytes, which act as supporting glial cells; and a fenestrated capillary network, which facilitates the release of hormones into the bloodstream [1,2].

The neurohypophysis stores and releases two essential hormones [3]. Arginine vasopressin (AVP) or antidiuretic hormone (ADH) regulates water reabsorption in the kidneys, preventing excessive fluid loss and maintaining water balance. It also contributes to blood pressure control by vasoconstriction. Its deficiency causes central diabetes insipidus (AVP deficiency, AVPD syndrome), with polyuria and polydipsia. Oxytocin induces uterine contractions in labor and facilitates the ejection of breast milk. It is also involved in the modulation of social behavior and bonding.

Posterior pituitary tumors (PPTs) are rare non-neuroendocrine neoplasms that originate in the neurohypophysis or its supporting structures [4]. Unlike adenohypophyseal tumors, they are not usually associated with hormonal hypersecretion, but may cause neuroendocrine dysfunction or compressive effects on adjacent structures. Since these tumors can compromise the production and release of AVP, a characteristic symptom of their presence is the appearance of central diabetes insipidus, characterized by polyuria and polydipsia. In addition, they can generate neurological symptoms due to compression of the optic chiasm or neighboring structures.

According to the 2025 World Health Organization (WHO) classification of pituitary tumors [5], PPTs are now grouped as low-grade neoplasms derived from pituicytes, comprising four histopathologically defined subtypes: pituicytoma, granular cell tumor of the sellar region (or granular cell pituicytoma), spindle cell oncocytoma (or oncocytic pituicytoma), and ependymal pituicytoma (previously known as sellar ependymoma). These tumors share diffuse expression of thyroid transcription factor 1 (TTF-1), confirming a common origin from the posterior pituitary, and differ in morphology, immunophenotype, and ultrastructural characteristics.

PPTs are infrequent and poorly studied entities, which makes their diagnosis and management difficult. In recent years, new studies have been published that have provided relevant information on their epidemiology, etiopathogenesis, clinical manifestations, diagnostic strategies, therapeutic approaches, and prognosis. However, challenges persist in their early identification, differentiation from other pituitary lesions, and selection of the optimal treatment. Therefore, an updated review is essential to analyze the latest evidence, identify advances in diagnosis and therapy, and evaluate the limitations and challenges still present in the management of these tumors.

## 2. Methods

The Medical Subject Headings (MeSH) terms used for the search included “posterior pituitary tumors”, “pituicytoma”, “granular cell tumor”, “spindle cell oncocytoma”, and “sellar ependymoma”. These terms were used to search PubMed/Medline, Cochrane Database of Systematic Reviews, and Embase. The most relevant articles in English were selected and reviewed, with the search covering articles preferably published within the last 5–10 years and up until 30 April 2025.

The inclusion criteria comprised original studies, narrative and systematic reviews, and meta-analyses that evaluated posterior pituitary tumors. Studies focusing on the histopathological types, clinical presentation, imaging studies, treatment strategies, and prognosis were also included. Abstracts and non-English articles were excluded.

## 3. Classification and Nomenclature of Posterior Pituitary Tumors

PPTs can be classified into primary and metastatic tumors (mainly breast and lung cancer). Primary tumors of the PPTs are low-grade non-neuroendocrine neoplasms that originate in the sellar and parasellar region, sharing common histopathologic and molecular features, including positivity for thyroid transcription factor 1 (TTF-1).

According to the 2017 World Health Organization (WHO) classification, four main types are included: pituicytoma (PTC), a tumor derived from pituicytes, which are specialized glial cells of the neurohypophysis; granular cell tumor (GCT), which is thought to arise from granular pituicytes; spindle cell oncocytoma (SCO), considered a variant of pituicytoma with distinctive histologic features; and sellar ependymoma (SE), a rare tumor that can also originate in the neurohypophysis region [6,7,8].

However, the classification of PPTs is still a matter of debate [4]. In the most recent update published in March 2025 by the World Health Organization [5], pituicytomas are defined as a distinct group of related low-grade neoplasms composed of pituicytes from the posterior pituitary and infundibulum. This classification includes four recognized histological subtypes: pituicytoma (ICD-O 9432/1), granular cell tumor of the sellar region/granular cell pituicytoma (9582/0), spindle cell oncocytoma/oncocytic pituicytoma (8290/0), and ependymal pituicytoma (9391/1). All share ubiquitous nuclear TTF-1 expression and lack neuroendocrine or adenohypophyseal differentiation markers. These entities are considered part of a histogenetically unified spectrum, supported by molecular and epigenetic studies, though they retain distinct morphological and clinical profiles. The WHO now recommends the use of unified terminology (e.g., “pituicytoma with granular/oncocytic/ependymal features”) and discourages previous designations such as posterior pituitary astrocytoma, spindle cell oncocytoma of the anterior pituitary, or Abrikossoff tumor.

## 4. Epidemiology

PPTs are rare [7], with an estimated prevalence of less than 1% of all pituitary tumors [9], and are detected in about 8.7% of pituitary autopsies [10]. Fewer than 400 cases have been reported to date [7,11]. PPTs usually occur in adults, with a peak incidence between the ages of 50 and 60. Patients with SCO tend to be somewhat older, with a mean age of 61.6 years, and no significant predominance between men and women has been observed [4,6,7,9]. To date, only one case of pituicytoma during pregnancy has been reported [12].

Recent studies have identified genetic mutations in the MAPK/PI3K pathways, such as FGFR1, HRAS, BRAF, NF1, among others, that are present in a significant proportion of these tumors, especially in PTC and SCO. In addition, loss of heterozygosity in certain chromosomes, such as 1p and 13q, has also been associated with these tumors [13,14]. The association with adenohypophyseal hyperfunction syndromes, such as Cushing’s disease and acromegaly, has been documented in several cases. This relationship suggests that hormonal dysfunction may play a role in the pathogenesis of these tumors [15]. Although PPTs can affect individuals of all ages and sexes, some studies have shown a slight predominance in women and older individuals [16,17].

**Figure 1:**
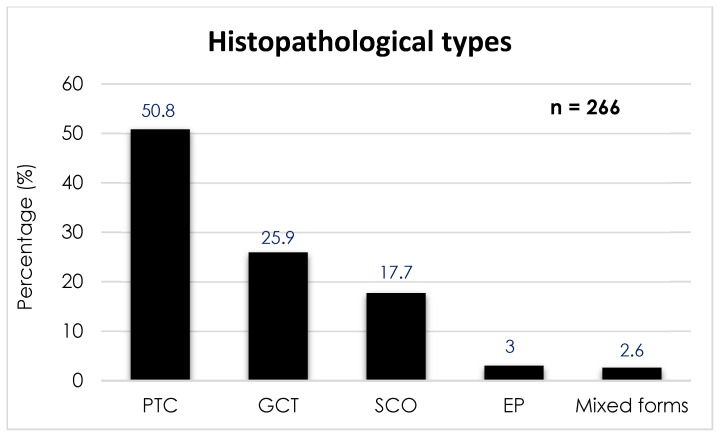
Prevalence of the different histopathological types of posterior pituitary tumors. Abbreviations: PTC, pituicytoma; GCT, granular cell tumor; SCO, spindle cell oncocytoma; EP, ependymal pituicytoma.

According to the systematic review of Guerrero-Pérez et al. [7] published in 2019 and conducted on 266 patients, the prevalence of the different types of PPTs is distributed as follows (Figure 1): PTC is the most common, with 135 cases (50.8%); followed by GCT with 69 cases (25.9%); SCO with 47 cases (17.7%); SE with 8 cases (3.0%); and, finally, tumors of mixed histology with 7 cases (2.6%).

## 5. Histopathological Types

The current classification and main histologic features of posterior pituitary tumors are summarized in Table 1.

## 6. Pituicytoma (PTC)

PTC, also known as classical pituicytoma, was first described in 1955 [18], but its diagnostic criteria were not established until 2000 by Brat et al. [19]. The WHO recognized it as a distinct entity in its third edition in 2007. Their diagnosis is based on histopathologic and immunohistochemical features. PTC is composed of spindle-shaped or slightly oval cells arranged in fascicles or storiform patterns, with fibrillar cytoplasm and oval to elongated nuclei containing small nucleoli. A sparse perivascular reticulin network is a characteristic histological finding. These tumors lack Rosenthal fibers, eosinophilic granular bodies, and Herring bodies, which helps differentiate them from other glial or neurohypophyseal lesions [4,19,20,21,22,23].

**Figure 2:**
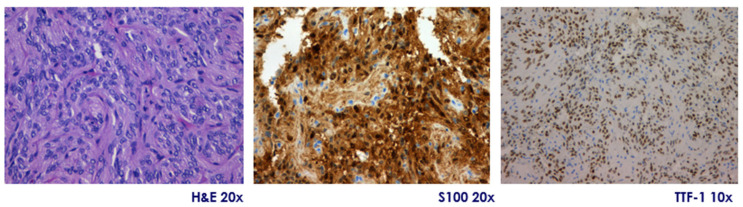
Pituicytoma. Representative microphotographs showing tumor histology with hematoxylin-eosin (H&E, original magnification 20×) staining showing a well-demarcated proliferation of spindle cells arranged in compact fascicles, with little pleomorphism and no evident mitotic activity, diffuse positivity for S100 protein (immunohistochemistry, 20×), and nuclear expression of TTF-1 (immunohistochemistry, 10×). Courtesy of Dr. Héctor Pian, Pathological Anatomy Department, Hospital Ramón y Cajal, Madrid, Spain.

They are immunopositive for S-100, vimentin, and show diffuse nuclear expression of TTF-1, a hallmark of posterior pituitary origin. Expression of GFAP is variable, and immunostaining is consistently negative for pituitary hormones, pituitary-specific transcription factors, chromogranin A, and synaptophysin, excluding adenohypophyseal neuroendocrine tumors (PitNETs). Their proliferation index (Ki-67) is typically low (<3%), but occasional cases exceed 5%, a finding that may correlate with more aggressive histological behavior. Atypical variants have been described, showing increased cellularity, pleomorphism, mitotic activity, and higher Ki-67 indices. According to the 2025 WHO classification, pituicytomas are included within a spectrum of pituicyte-derived tumors with shared TTF-1 positivity and common ontogeny from ventral neuroectoderm. Molecular studies have identified alterations in the MAPK pathway (e.g., *HRAS*, *BRAF*, *FGFR1*), and most tumors show positive immunoreactivity for phosphorylated ERK, supporting pathway activation and providing a potential rationale for targeted therapy [24] (Figure 2).

## 7. Granular Cell Tumor (GCT)

GCT, also referred to as granular cell tumor of the sellar region or granular pituicytoma according to the 2025 WHO classification, is composed of polygonal to ovoid cells with abundant, eosinophilic, granular cytoplasm that is PAS-positive and diastase-resistant, reflecting lysosomal accumulation. The architectural pattern may vary, typically showing nodular, layered, and/or fascicular arrangements. Tumor nuclei are usually small with inconspicuous nucleoli, although nuclear atypia may be mild to marked. Mitoses are rare, and necrosis is uncommon; however, atypical forms have been reported, showing increased nuclear atypia and mitotic figures.

According to the 2025 WHO classification, GCTs are recognized as one of the four subtypes of posterior pituitary tumors derived from pituicytes, unified by diffuse nuclear TTF-1 expression and a shared origin from ventral neuroectoderm. Immunohistochemically, GCTs are positive for TTF-1, S-100 protein, vimentin, GFAP (variably), CD68, alpha-1-antitrypsin, cathepsin B, and Leu7 [4,7]. Electron microscopy confirms the cytoplasmic lysosomal nature, revealing numerous polymorphic lysosomes of variable electron density, which correlate with the granular appearance seen in light microscopy. Notably, secretory granules are absent, distinguishing GCTs from neuroendocrine neoplasms of the adenohypophysis [25,26,27,28]. On ultrastructural examination, the cytoplasm appears to be packed with lysosomes, while secretory granules are absent [4]. This entity replaces outdated terms such as Abrikossoff tumor, granular cell myoblastoma, granular cell neuroma, and granular cell schwannoma, which are no longer recommended under current WHO terminology.

## 8. Spindle Cell Oncocytoma (SCO)

SCO, now referred to as oncocytic pituicytoma in the 2025 WHO classification, is composed of spindle-shaped cells with granular, eosinophilic cytoplasm due to the accumulation of abundant abnormal mitochondria, conferring the characteristic oncocytic appearance. The presence of small and sparse neurosecretory granules may also be observed, although it is not a constant feature. Nuclei are typically moderate in size and may display mild to moderate nuclear atypia. Mitoses are usually rare, and the Ki-67 proliferation index is generally low, though cases with significant mitotic activity and elevated Ki-67 values (>5%) have been reported, which may be associated with increased risk of recurrence [4,29].

According to the 2025 WHO classification, SCO represents one of four recognized subtypes of posterior pituitary tumors derived from pituicytes, unified by diffuse nuclear TTF-1 expression and a shared origin from the ventral neuroectoderm. Immunohistochemically, tumor cells are positive for vimentin, S-100 protein, TTF-1, galectin-3, annexin A1, somatostatin receptors, and show diffuse or focal membrane staining for epithelial membrane antigen (EMA). They are consistently negative for GFAP, cytokeratins, chromogranin A (CgA), and pituitary hormones, allowing distinction from adenohypophyseal (PitNET) tumors [4,14,29,30,31,32].

These immunophenotypic and ultrastructural features support the classification of SCO as a pituicyte-derived oncocytic neoplasm, rather than an epithelial or neuroendocrine tumor.

## 9. Ependymal Pituicytoma (EP)

EP, previously referred to as sellar ependymoma, is a rare neoplasm, with fewer than 20 cases reported to date. According to the 2025 WHO classification, it is now recognized as a distinct ependymal variant within the family of PPTs, all of which are derived from pituicytes and characterized by nuclear TTF-1 expression and lack of pituitary hormone production.

Histologically, EP is composed of sheets and fascicles of spindle-shaped to epithelioid cells with scant cytoplasm and rounded to oval nuclei. Characteristic ependymal features include perivascular pseudorosettes, true ependymal rosettes, and ependymoma-like follicular structures [33,34,35,36]. Nuclear atypia is variable, and mitoses are generally rare. Immunohistochemically, EP shows variable expression of GFAP, vimentin, and S-100 protein, and epithelial membrane antigen (EMA) typically demonstrates a paranuclear dot-like, apical, or luminal membranous pattern, consistent with ependymal differentiation. Additional immunoreactivity may be observed for CAM 5.2, CD99, Bcl-2, and galectin-3, although these are inconsistently expressed and not specific. Ultrastructurally, tumor cells show intra- and intercellular microlumina lined with microvilli and occasional cilia, along with intermediate filaments, microtubules, and well-formed cell junctions, supporting their ependymal phenotype [4,6].

Due to its rarity and overlapping features with other sellar lesions, accurate diagnosis of EP requires integration of histopathologic, ultrastructural, immunohistochemical, and radiological findings, with particular attention to distinguishing it from other members of the PPT spectrum and central nervous system ependymomas.

## 10. Clinical Presentation

PPTs can manifest with a wide variety of symptoms, mainly due to compression of adjacent structures or hormonal dysfunction, these factors being mainly influenced by the size and location of the tumor. Patients with suprasellar PPTs usually present with isolated visual symptoms due to optic nerve compression, whereas intrasellar tumors more frequently affect the pituitary gland and adjacent structures, causing headache and hypopituitarism.

### 10.1. Visual Disturbances

Among the most common manifestations are visual disturbances, with an overall prevalence of 48.8% to 58.1% [7,11]. Regarding the different types of PPTs, visual defects were observed in 50.4% of patients with PTCs (68 of 135), 55.1% of those with GCTs (38 of 69), and 61.7% in cases of SCOs (29 of 47). Although SCOs exhibited the highest frequency of visual disturbances, followed by GCTs and PTCs, no significant differences were found among the three tumor types [7].

### 10.2. Headache

Headache is the second most common symptom associated with PPTs, with a prevalence of 25.6% to 40.5%. It is typically caused by the tumor’s local pressure on adjacent structures and often improves after surgical resection of the tumor [7,11]. No significant differences have been found in the prevalence of headache among the three most common forms of PPTs, affecting approximately 35% of each group [7].

### 10.3. Hypopituitarism

The presence of hypopituitarism has been reported in 21.8% to 37.5% of patients [7,11,37,38]. Although gonadotropin deficiency is the most common hormone deficiency, other deficiencies have also been identified, including a case of isolated ACTH deficiency. PPTs often cause mild hyperprolactinemia [38]; however, few cases associated with galactorrhea have been reported [7].

AVPD syndrome is rare at the time of diagnosis in patients with PPT, despite the neurohypophyseal origin of these tumors, with a prevalence of 1.5% to 5% [7,38,39]. Their slow growth, unlike metastases, makes degeneration of more than 80–90% of the AVP-secreting neurons in the hypothalamic supraoptic and paraventricular nuclei, a necessary threshold for developing diabetes insipidus before the tumor is clinically detected by its mass effects, unlikely. However, after surgery, its prevalence increases considerably, affecting approximately 18–34% of patients transiently or permanently, without differences between the different histological types, making it a frequent complication of surgical [7].

### 10.4. Adenohypophyseal Hyperfunction Syndromes

In recent years, cases of adenohypophyseal hyperfunction syndromes have been documented, preferably of ACTH (Cushing’s disease), GH (acromegaly), and hyperprolactinemia associated with PPTs [11,15,40]. Although few cases have been reported so far, the association of these tumors with anterior pituitary-dependent hormone hypersecretion syndromes seems to be more frequent than would be expected by simple coincidence. The published series point out certain distinctive features, such as a higher prevalence in women, the predominance of PTCs, a smaller size of PPTs compared to sporadic cases, and their incidental detection in all patients. Although surgical cure rates of PPTs have been described as high, in most cases, no secretory adenoma was identified on histopathologic study. However, remission of the hormone hypersecretion syndrome was not always achieved, particularly in patients with acromegaly [15].

#### 10.4.1. Cushing’s Disease

ACTH-dependent hypercortisolism associated with PPTs has a reported prevalence of approximately 5–6%, with most cases being PTCs, followed by GTCs [15,40]. More recently, two cases of SCO have been documented [11,41]. To date, no instances of Cushing’s disease linked to SE have been identified. Given that Cushing’s disease is a rare condition, with an incidence of 6.2–7.6 cases per million person-years [42], the 5–6% prevalence observed in PPT patients is notably higher than expected in the general population. This discrepancy suggests that the coexistence of both conditions is unlikely to be coincidental and may indicate an underlying pathophysiological connection favoring the development of hypercortisolism in this context. Cushing’s disease associated with PPTs predominantly affects women (2.5:1 ratio), with a mean age at diagnosis of 41 years (range: 7–62 years). Symptoms include hypertension, central obesity, and alterations in cortisol dynamics. In 60% of patients, no ACTH-producing histological lesions were identified in the surgical specimens, while positive cases showed corticotroph adenomas or corticotroph hyperplasia. After surgery, 66% showed remission of Cushing’s disease, regardless of histological findings. Cushing disease-related tumors are usually microadenomas (<10 mm) and are often detected incidentally in histopatologic studies [15].

#### 10.4.2. Acromegaly

Although less frequently than in Cushing’s disease (CD), cases of PPT and acromegaly have also been documented [7,15,40,43]. To date, only about ten cases of acromegalic patients associated with PPT have been reported, all in the last 15 years, suggesting a prevalence of 2.3% [15]. Most of these cases (66.7%) corresponded to PTC, while the rest were GTC. No cases of acromegaly associated with either SCO or SE have been described to date. Although this prevalence may seem low, considering the low incidence of acromegaly in the general population (2.8–13.7 cases per 100,000 population) [44] and the small number of documented PTC (less than 400 cases), this association is not considered to be fortuitous.

The characteristics of patients with acromegaly associated with PPTs are summarized as follows: six patients were described, five of whom were women (83.3%), with a mean age of 51.3 years (±15.3) and an age range between 29 and 79 years. Regarding tumor type, four cases corresponded to PTC (66.7%) and two to GTC (33.3%). The average maximum diameter of the tumors was 14.4 mm (±3.5). All PPTs were incidental findings. In three patients (50%), a total resection was performed, and, in the other three (50%), a subtotal resection was performed. After the first surgery, cure of the PPT was achieved in five cases (83.3%), while, in one case, the tumor persisted (16.7%), with no recurrences reported. As for the control of acromegaly, it persisted in four patients (66.6%), and remission was achieved in two (33.3%) [15].

#### 10.4.3. Hyperprolactinemia

Hyperprolactinemia is the most frequent hormonal disorder of the anterior pituitary associated with PPTs, present in approximately 40% of cases. It is usually mild, although cases accompanied by galactorrhea have been reported. So far, no prolactinomas associated with PPT have been documented with histologic confirmation [15]. Generally, hyperprolactinemia is mild and is assumed to be due to compression of the pituitary stalk, rather than to directal prolactin production due to the lesion or the coexistence of a prolactinoma. [45]. The absence of confirmed prolactinomas in association with PPT could be explained by the fact that surgery is rarely indicated in cases of mild hyperprolactinemia or adenomas, since these patients usually respond adequately to treatment with dopaminergic agonists.

#### 10.4.4. Pathophysiological Mechanisms

The relationship between PPTs and adenohypophyseal hypersecretion syndromes is not fully elucidated, and several mechanisms have been proposed to explain this association [15,40]. PPTs could induce endocrine hyperfunction by hypersecretion of ACTH or GH through local or systemic mechanisms. In addition, their compression on the pituitary stalk may reduce dopaminergic inhibitory signaling, favoring hyperprolactinemia. It has also been proposed that these tumors could stimulate the production of hypothalamic hormones such as CRH or GHRH, which indirectly potentiates ACTH or GH secretion. Likewise, the release of cytokines and growth factors by PPTs could induce hyperplasia or tumorigenesis in the adenohypophysis. Another hypothesis suggests a common cellular origin between PPTs and anterior pituitary tumors, which would explain their coexistence. However, in some cases, the simultaneous presence of both conditions could be merely coincidental, without a direct pathophysiological mechanism linking them [15,40].

## 11. Radiographic Features

Sellar magnetic resonance imaging (MRI) with high-resolution protocols is the imaging modality of choice for diagnosing PPTs. The routine MRI protocol for evaluating the sellar region consists of T1-weighted spin-echo sequences acquired in coronal and sagittal planes, both before and after the administration of a gadolinium-based contrast agent, along with coronal T2-weighted fast spin-echo sequences [46].

PPTs lack distinctive radiological findings, which has led to their systematic misdiagnosis as anterior pituitary adenomas or other lesions located in the sellar or suprasellar region, such as meningiomas, craniopharyngiomas, hypophysitis, or metastases [7,16].

**Figure 3:**
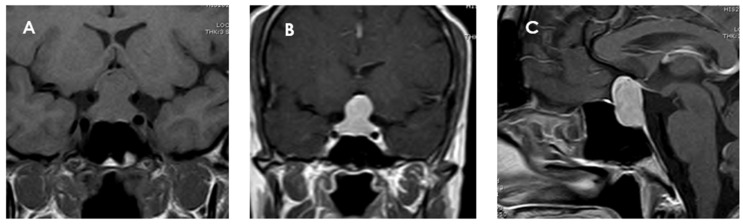
Magnetic resonance imaging of a pituicytoma (PTC). The lesion measures 25 × 12 × 14 mm (cranial–caudal × anteroposterior × transverse), with superior displacement of the optic chiasm and discrete compression of the pituitary stalk. (**A**) Coronal T1 sequence without contrast showing an isointense mass located in the sellar and suprasellar region. (**B**) Coronal T1-weighted coronal section with gadolinium showing homogeneous enhancement of the lesion. (**C**) Sagittal T1-weighted contrast-enhanced sagittal section confirming the suprasellar location of the mass, well delimited and with intense contrast uptake, and with no signs of invasion to adjacent structures.

PPTs usually present as well-demarcated solid masses, located in the sellar and suprasellar region or confined exclusively to that region. In T1-weighted sequences, PTCs are mostly round or oval, sharply defined, and located in the sellar and/or suprasellar region. Tumors are generally isointense (79%) to gray matter on T1-weighted images and isointense to slightly hyperintense on T2-weighted images, although these characteristics may vary according to the cellular composition of the tumor [16,39,47,48,49]. A significant portion (74%) of PTCs are homogeneously enhanced on MRI after gadolinium contrast administration, suggesting a well-developed capillary network (Figure 3). This contrast uptake pattern distinguishes them from GCTs and SCOs, which often show heterogeneous enhancement [39]. Although most are solid lesions, in a small percentage of cases (about 2%), cystic components have been identified.

**Figure 4:**
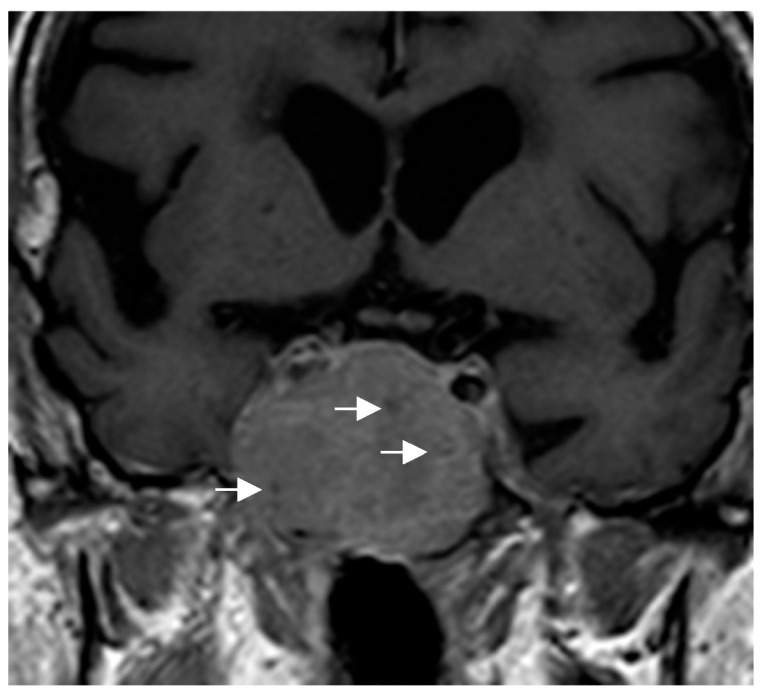
Coronal T1-weighted image showing an intra-suprasellar spindle cell oncocytoma (SCO). Multiple hypointense millimetric foci (white arrows) are seen in the lesion.

Because of their size and location, they can exert a mass effect on adjacent structures, including compression of the optic chiasm. Some authors have proposed specific radiological features that could guide the diagnosis: in PTC, this possibility should be considered in masses centered in the suprasellar region [50], while in GCTs, the diagnosis is suggested for very suprasellar or intra-suprasellar masses in which the posterior lobe of the pituitary is not identified on imaging [38,51]. In addition, GCTs may exhibit a characteristic pattern known as a “star-like crack”. This pattern is described as a series of linear structures that become convergent shapes, which appear as if they were cracks or lines radiating from a central point, giving the impression of a star [17]. With respect to SCO, the presence of multiple millimetric hypointense foci and linear T1 signal void areas (probably hemosiderin deposits and vascular structures, respectively) has been proposed as the most important distinctive radiological finding of SCO and may help to presuppose the diagnosis [52] (Figure 4). Given the small number of cases described, there is no information available to identify specific radiological characteristics of SE. In general, PPTs do not infiltrate the adjacent cavernous sinuses nor are they associated with significant cerebral edema, unlike other tumors in the region.

The differentiation between PPTs and non-functioning pituitary adenohypophyseal tumors (NF-PitNETs) is important because of the implications for surgical planning and perioperative management. PPTs, because of their location and anatomical characteristics, require special attention to the pituitary stalk and hypothalamus during surgery to minimize the risk of severe neuroendocrine dysfunction. In this context, a recent article evaluated the preoperative prediction of NF-PitNETs and PPTs by analyzing radiomic features extracted from MRI images. The study included images from 110 patients with NF-PitNETs and 55 with PPTs, and developed an individualized predictive model based on logistic regression, which integrated radiomic features and clinical data. The results showed that the model achieved an accuracy of 87.9%, a sensitivity of 93.6%, and a specificity of 76.4%, significantly exceeding the discriminative ability of isolated clinical features. The incorporation of radiomic signatures as noninvasive imaging biomarkers could represent an important advance in the accurate classification of pituitary tumors, contributing to improving preoperative preparation, optimizing surgical strategy, and reducing preoperative diagnostic errors [53].

Although MRI is the preferred modality, computed tomography (CT) may be useful in certain situations. PTCs may present as hyperdense masses compared to the brain parenchyma on CT images and usually show homogeneous enhancement after contrast administration [39].

## 12. Treatment Strategies

The main therapeutic strategies in PPTs are surgery and radiotherapy; however, in the future, targeted medical therapies could be incorporated into the approach to these tumors.

### 12.1. Surgery

Surgery is the treatment of choice for PPTs, and total resection represents a cure in most cases. However, the firmness of the tumor, its adherence to adjacent structures, and its high vascularity often make complete removal difficult. In some series, tumor persistence was observed in 45% to 66% of patients with available follow-up [7,17].

The most commonly used approach is transsphenoidal surgery (63%), although in some cases, a second intervention by craniotomy is required. The latter is considered preferable in tumors with significant suprasellar or parasellar extension, or after failure of the transsphenoidal approach. Visual impairment rates after surgery are highly dependent on the location and extent of the tumor. Indeed, tumors with suprasellar or retroinfundibular extension may present limitations for complete resection by standard endonasal approach, given the restricted visualization angle to the optic chiasm and superior structures. In these cases, craniotomy—although associated with a higher risk of morbidity—may allow a more direct visualization of the optic nerve and facilitate a more complete resection in certain locations. In contrast, the standard transsphenoidal approach presents less risk of visual sequelae, but may be limited by orientation and access to the tumor, resulting in a higher rate of subtotal resection, especially in lesions with suprasellar growth or firmly adherent to the hypothalamus or pituitary stalk [7].

Reports of PPTs located in the retroinfundibular or superior suprasellar regions have highlighted the limitations of anterior surgical approaches in achieving complete and safe tumor resections. In this specific anatomical context, the posterior transpetrosal approach has been recently proposed as a valuable alternative, minimizing the risk of damage to critical structures along the pathway [54].

Postoperative complications after PPT surgery are frequent and, in some cases, severe. The most common is hypopituitarism, present in approximately 42% of patients, with involvement of one or more hormonal axes. It is followed by AVPD syndrome, temporary or permanent, which occurs in about 33% of cases [7]. Intraoperative hemorrhage represents a critical complication, observed in about 30% of patients, although some recent series report rates of up to 75% [7,55]. Cerebral angiography has been used occasionally, mainly in situations of severe hemorrhagic complications or radiological findings suggestive of vascular pathology. Other relevant complications include cerebrospinal fluid (CSF) leak (2.6%); syndrome of inappropriate antidiuretic hormone secretion (SIADH), observed in approximately 2% of cases and associated with hyponatremia; and meningitis, which occurs in about 1%, generally secondary to CSF leaks. Neurological complications have also been reported, such as visual loss, cranial nerve palsies, hemiparesis, hydrocephaly, diencephalic syndrome, deep vein thrombosis, and, in exceptional cases, cardiogenic shock or pseudoaneurysm formation. It should be noted that the real rate of complications may be underestimated, since, in some series, information was missing in up to 40% of the cases. Therefore, close and multidisciplinary follow-up is essential for the early detection and management of these complications [7].

### 12.2. Radiotherapy

The therapeutic role (indication, type, and usefulness) of radiotherapy is not clearly established in the management of PPTs. The use of radiotherapy as a primary or adjuvant treatment is controversial and is not routinely recommended. Radiotherapy may be considered in cases with aggressive features, progressive disease, or inoperable recurrence [17,56]. It has been used in approximately 25% of patients with persistent or recurrent disease, although the type of technique used is often not specified [7]. In some recent cases, stereotactic radiotherapy or radiosurgery has been used with variable results: some patients show tumor stability during follow-up ranging from 3 months to 7 years, while in other cases, tumor progression has been observed despite treatment. Moreover, in many cases, neither the response nor the follow-up time after radiotherapy is detailed, which limits the interpretation of its real efficacy [7,17,57].

### 12.3. Potential Molecular Targets

Targeted therapies for PPTs remain limited, primarily due to their low incidence and the scarcity of large-scale clinical studies. Nevertheless, recent advances in molecular profiling have identified actionable targets that may guide future therapeutic strategies, particularly in non-resectable or recurrent cases.

In PTCs, immunohistochemical and transcriptomic studies have shown expression of vascular endothelial growth factor (VEGF) receptors, as well as consistent membranous expression of somatostatin receptors SSTR3 and SSTR5. These findings suggest a potential role for VEGFR-targeted therapies (e.g., bevacizumab) and somatostatin analogs (e.g., pasireotide) in selected patients with residual or progressive disease [58].

More significantly, next-generation sequencing and methylation-based profiling have revealed recurrent somatic alterations in key signaling pathways, particularly the MAPK and PI3K pathways, in subsets of PTCs and SCOs. Reported mutations include alterations in FGFR1, HRAS, BRAF, NF1, CBL, MAP2K2, and TSC1 [13,24,59,60,61]. These findings align with the 2025 WHO classification, which acknowledges the molecular overlap among pituicyte-derived tumors despite their histological differences.

As a result, emerging targeted therapeutic options for these tumors may include BRAF inhibitors (e.g., dabrafenib, vemurafenib), MEK inhibitors (e.g., trametinib), and other agents directed against MAPK/PI3K pathway effectors. While clinical data remain preliminary, such treatments could be considered in selected patients within the context of molecular profiling and clinical trials.

## 13. Prognosis

The prognosis of PPTs varies depending on factors such as histological subtype, tumor size, anatomical location, clinical presentation, and therapeutic response. In general, tumor-specific survival at 5 years approaches 100%, reflecting an overall favorable prognosis with respect to tumor-related mortality [16]. However, tumor recurrence is observed in approximately 16% of cases, often requiring adjuvant radiotherapy for disease control [14,16]. Recurrence is particularly more frequent in SCOs, which are noted to have greater vascularity, firm consistency, and adhesiveness to adjacent structures, often precluding complete surgical excision—a point emphasized in the 2025 WHO classification [62,63].

EPs exhibit a more variable clinical course, largely influenced by tumor size, anatomical extension, and the feasibility of complete resection. Larger tumors or those infiltrating critical structures (e.g., cavernous sinus, optic apparatus) pose surgical challenges and may be associated with worse outcomes. Beyond oncologic control, postoperative complications such as hypopituitarism, central diabetes insipidus (AVP deficiency syndrome), and visual impairment can significantly affect long-term quality of life and functional prognosis. While these tumors are histologically low-grade, their potential for morbidity and recurrence supports the need for close, long-term multidisciplinary follow-up [16,17].

The WHO 2025 update reinforces the importance of stratifying prognosis based not only on histological subtype but also on emerging molecular profiles, particularly in tumors with documented copy number alterations or MAPK/PI3K pathway activation. A coordinated, multidisciplinary approach—integrating neurosurgery, endocrinology, pathology, radiation oncology, and molecular diagnostics—is essential to optimize clinical outcomes and reduce long-term morbidity.

## 14. Conclusions

In conclusion, PPTs are rare entities with remarkable histologic and clinical heterogeneity. Although their behavior is usually indolent, they can cause compressive symptoms and significant endocrine dysfunction. Diagnosis remains a challenge due to their low prevalence and the absence of specific radiological findings. Surgery is the treatment of choice, although it is often associated with complications. The recognition of molecular alterations and the expression of specific receptors opens new avenues for targeted therapies. Further studies are needed to improve early diagnosis, optimize treatment, and define long-term follow-up strategies.

## Figures and Tables

**Table 1:** Updated histologic classification of posterior pituitary tumors (PPTs) according to the 2025 WHO classification.

Tumor Type	Cellular Origin	Characteristic Histology	Immunohistochemical Markers	TTF-1 Expresión
Pituicytoma (PTC)	Pituicytes (light/dark subtypes)	Spindle or bipolar cells in short fascicles or storiform pattern; low mitotic index	S-100, vimentin, ±GFAP; negative for cytokeratins and PitNET markers	Diffuse positive
Granular cell tumor (GCT)	Granular pituicytes	Polygonal cells with eosinophilic granular PAS+ and diastase-resistant cytoplasm	CD68, S-100, vimentin, α1-antitrypsin, ±GFAP	Diffuse positive
Spindle cell oncocytoma (SCO)	Oncocytic pituicytes	Spindle to epithelioid cells with eosinophilic cytoplasm rich in mitochondria	S-100, galectin-3, antimitochondrial antibody, EMA, ±GFAP	Diffuse positive
Ependymal pituicytoma (EP)	Ependymal-like pituicytes	Rosettes, perivascular pseudorosettes; no necrosis; rare mitoses	S-100, EMA (dot-like/apical), vimentin; ±GFAP	Diffuse to focal

## Data Availability

No original data are associated with this manuscript.
